# Trans Fat Labeling Information on Brazilian Packaged Foods

**DOI:** 10.3390/nu11092130

**Published:** 2019-09-06

**Authors:** Camila Zancheta Ricardo, Isabela Mateus Peroseni, Laís Amaral Mais, Ana Paula Bortoletto Martins, Ana Clara Duran

**Affiliations:** 1 Center for Epidemiological Research in Nutrition and Public Health, University of Sao Paulo, Sao Paulo 01246-904, Brazil (A.P.B.M.) (A.C.D.); 2 Center for Food Studies, University of Campinas, Campinas 13083-970, Brazil; 3 Brazilian Institute for Consumer Defense, Sao Paulo 05002-050, Brazil

**Keywords:** trans-fatty acids, ultra-processed food products, food labels, consumer, non-communicable disease

## Abstract

Although the adverse effects of trans fat consumption are well documented, industrially-produced trans fats are still used in a variety of food products. Our objective was to investigate the presence of trans fat information on the nutrition facts panel, in the list of ingredients, and the use of trans fat claims in packaged food and beverages marketed in Brazil. This was a cross-sectional study that used data from packaged food and beverages available in the five supermarket chains with the largest market share in Brazil. Of the 11,434 products that were analyzed, 81.3% did not present a source of trans fats in the list of ingredients. The percentages of products with specific (hydrogenated fats or oils) and unspecific trans fat terms (margarine, vegetable fat, and vegetable cream) in the list of ingredients were 4.1% and 14.6%, respectively. Bakery products, cookies and crackers, candies and desserts, snacks, and convenience foods had the highest percentages of trans fat claims. We also found claims in products with ingredients that are sources of trans fats. In conclusion, trans fat ingredients were found in almost one-fifth of the Brazilian packaged foods. The current Brazilian legislation is not sufficient to inform consumers about the content of trans fats in packaged foods. Along with measures to restrict the use of industrially-produced trans fats, improvements in nutritional labeling are also needed.

## 1. Introduction

Trans fats are unsaturated fatty acids with at least one double bond in the trans configuration. They occur naturally at low levels in meat and dairy products from ruminants and are industrially produced by the partial hydrogenation of vegetable oils [1]. Trans fats are attractive to the food industry because they increase the shelf life of foods and oils and improve taste and texture at a low cost. Therefore, a variety of ultra-processed food products (UPP), such as bakery products, cookies and crackers, snacks, sweets, ice cream, and ready-to-eat and frozen foods, contain trans fats [2,3,4,5].

The adverse health effects of trans fat consumption, particularly the association with increased risk of coronary heart disease (CHD), are well documented in the literature [1,6,7]. Although there is a clear relationship between the intake of trans fats and the risk of CHD, ruminant trans fats have no apparent health effects, which is likely due to the low content in the diet of the general population [8]. Furthermore, no benefits for health or “safe level” of trans fat intake has been identified. Compounding this issue, the consumption of trans fats exceeding the maximum limit proposed by the World Health Organization (WHO) (1% of total energy intake) [9] occurs mainly because of the availability of industrially-produced trans fats [10].

Over the past decades, voluntary and regulatory measures to lower the content of industrially-produced trans fats, including improvements in food labeling, food reformulation, and restrictions in use, have been adopted around the world [11]. The most effective measures are policy interventions aimed at eliminating industrially-produced trans fats [4]. Denmark was the first country to restrict the use of trans fats by imposing a limit of two grams of trans fats per 100 g of fat or oil, which has virtually eliminated the consumption of trans fats in the country [12]. The restriction on the use of trans fat ingredients have been discussed in many countries worldwide and the WHO recommends the elimination of industrially-produced trans fats to reduce the burden of non-communicable diseases (NCDs) as one of their priority targets, which has guided the WHO’s strategic plan from 2019–2023 [13].

In 2003, the Brazilian Health Regulatory Agency (Agência Nacional de Vigilância Sanitária—Anvisa) included the information regarding the content of trans fats as a mandatory item on the nutrition facts panel of packaged foods and beverages labels in the country. However, the current legislation fails to provide clear and adequate information related to the content and presence of trans fats in packaged foods and beverages. First, trans fat content below 0.2 g per serving is considered insignificant and must be declared as zero on the nutrition facts panel [14]. Still, the portion sizes for different food groups are determined by legislation based on a recommended consumption, with small portion sizes for high caloric foods, such as biscuits and chocolates, [15], usually minor in comparison to the real portion people in Brazil eat [16]. Moreover, Brazilian regulations allow a variation of 20% in the nutrient content [14] and of 30% in the portion sizes [15] declared on the packages. Second, although the use of nutrition claims about trans fats are regulated in the country, it is possible that products with trans fat ingredients state a message such as “zero trans fats” or “trans fat free” if they present less than 0.1 g of trans fats per portion and also a low content of saturated fat (a maximum of 1.5 g of trans and saturated fats in a portion or per 100 g of prepared dishes and with less than 10% of energy from saturated fat) [17]. In addition to labeling rules, in 2007, the Brazilian Ministry of Health signed a voluntary agreement with food industry representatives to reduce trans fats in processed foods [18]. Despite these efforts, the consumption of trans fats in Brazil exceeds the limit proposed by WHO [10].

A review that investigated the consumption of trans fats in 29 countries worldwide found that in the majority of the studied countries, the trans fat intake is under the limit of 1% of total energy intake, and the main source of the trans fats consumed is from animal products [10]. However, data from the last survey about food consumption in Brazil conducted during the period 2008–2009 showed that the average trans fat intake in the country was equivalent to 1.4% of the total energy intake [19], and the main source of trans fats was industrial (e.g., oils and fats, biscuits, pizza, grains, seeds, nuts, chocolate, soups, savory snacks, meals, and restaurant foods) [10]. The scenario in the country is concerning since Brazil is facing changes in dietary pattern, with a crescent consumption of UPP [20], and the consumption of those products is associated with higher trans fat intake [21]. Analysis of a national survey showed that the quintile of Brazilians with lower UPP consumption was in line with the recommendation of trans fat (0.8% of total energy intake) and for those in the quintile with highest UPP consumption the trans fat intake was 1.9% of total energy intake, double that of those with lower UPP consumption and almost two times the limit established by WHO [19]. Moreover, in Brazil cardiovascular disease (CVD) is the leading cause of deaths [22], and it was estimated that more than 10% of the CHD deaths were due to high trans fat intake [23], equivalent to at least 10,000 deaths in the country in 2010 [24].

Considering the challenges of developing and implementing effective regulatory measures for the prevention and control of NCDs related to unhealthy diets in Brazil, the objective of the present study was to evaluate the presence of trans fat information and claims regarding trans fats in packaged foods and beverages marketed in Brazil.

## 2. Materials and Methods

### 2.1. Data Collection

This cross-sectional study used data from a sample of packaged foods and beverages that are sold in Brazilian supermarkets. Supermarkets were selected as the source for the data collection because they are the main places in which food is purchased in the country [25]. The five supermarket chains with the largest market share in Brazil were identified using food retail annual sales data from Euromonitor International in 2016 [26]. The primary study area was São Paulo, the largest Brazilian city, which is located in the Southeastern region of the country. Because one of the supermarket chains only had stores in the Northeastern region of the country, we collected data in Salvador, which is considered the largest city in the region. We obtained formal permission from all supermarket chains included in this study.

Trained fieldworkers photographed all sides of the packages of the products available in the stores between April and July 2017, according to the methods proposed by Kanter et al. (2017) [27]. We included approximately 13,000 different items. The product name, brand, flavor, package size, nutrition facts panel, and list of ingredients were entered by trained nutritionists in an online platform using a template developed by researchers from the Institute of Nutrition and Food Technology, University of Chile (Instituto de Nutrición y Tecnología de los Alimentos—INTA, Universidad de Chile, Santiago, Chile) and the University of North Carolina at Chapel Hill (UNC, Chapel Hill, NC, USA). We adapted and translated the template for this research.

We excluded duplicate records, products available in more than one package size, multipacks with different items, products without a nutrition facts panel, products without a list of ingredients, and products with missing values for portion size and/or calories. The final database was composed of 11,434 products. All products were included in the analysis of the nutrition facts panel and the list of ingredients.

Due to budget and time constraints, we performed the collection of claims information in a random subsample of 30% of the products (*n* = 3491). This was the first project aimed to identify the prevalence of nutrition claims in Brazil, so we guaranteed that our sample size was large enough to estimate even a small prevalence (*p* = 0.01) and its 95% confidence intervals (CI), considering a precision of 0.005 [28]. Claims collection and classification were based on the International Network for Food and Obesity/NCD Research, Monitoring and Action Support (INFORMAS) protocol [29]. In the present study, we considered the presence of trans fat claims in any part of the packages.

For analysis, we classified the food and beverages products in 25 categories presented in Appendix A.

### 2.2. Data Quality

For data on nutritional composition, 10% of the sample was entered twice by the same person, and 10% of the sample was entered by a second person for intra- and interrater reliability analysis. For data on claims, the entire subsample was entered twice: 50% for intra- and 50% for interrater reliability analysis. Intraclass correlation coefficients (ICC) were calculated for the content of trans fats, and Cohen’s kappa coefficients were calculated for the presence of claims. The ICC values were 0.905 and 0.949, and the kappa values were 0.949 and 0.893 for intra- and interrater, respectively. These analyses confirmed the reliability of the data [30]. We compared the proportion of food groups and the average nutrients in the database and the subsample, using chi-square and *t*-test, and found no differences.

### 2.3. Search for Terms in the List of Ingredients

Table 1 shows the keywords that were used to identify products with trans fat ingredients. Specific terms clearly identify ingredients that are a source of industrially-produced trans fats, but unspecific terms may or may not indicate a source of trans fats. Therefore, the latter terms are imprecise in determining the presence of industrially-produced trans fats in a product.

Based on the information of trans fats on the nutrition facts panel and in the list of ingredients, the foods were divided into four categories:

○ With trans fats: foods with information indicating a content of trans fats greater than zero on the nutrition facts panel;
○ False negatives: foods with no trans fats declared on the nutrition facts panel but with specific sources of trans fats in the list of ingredients;
○ Possible false negatives: foods with no trans fats declared on the nutrition facts panel but with unspecific sources of trans fats in the list of ingredients;
○ Without trans fats: foods with no specific or unspecific terms related to trans fats in the list of ingredients.

### 2.4. Statistical Analysis

We calculated the proportion and 95% confidence intervals (95% CI) of foods with information of trans fats on the nutrition fact panel, in the list of ingredients and with trans fat claims, by food categories. Additionally, we analyzed the proportion of the four aforementioned categories for the presence of trans fats in foods and beverages with claims.

## 3. Results

Of the 11,434 food products, 81.3% did not include ingredients that are sources of trans fats in the list of ingredients. The percentages of products with specific and unspecific ingredient sources of trans fats in the list of ingredients were 4.1% and 14.6%, respectively. Bakery products (20.7%), cookies and crackers (15.1%), snacks (12.6%), candies and desserts (8.2%), and convenience foods (6.0%) had the highest proportion of products with specific trans fat ingredients. Unspecific keywords in the list of ingredients were more prevalent in the same five food groups (Table 2).

Over 90% of the products reported zero or an insignificant amount of trans fats on the nutrition facts panel. Bakery products (17.6%), cheeses and cheese spreads (17.3%), convenience foods (16.7%), and cookies and crackers (13.0%) had the highest proportions of items with a listed content of trans fats above zero grams. Only a small portion of the products (1.4%) did not report the content of trans fats on the nutrition facts panel, and these products were mostly packaged fruits and vegetables, which are not a source of trans fats (Table 2).

**Figure 1:**
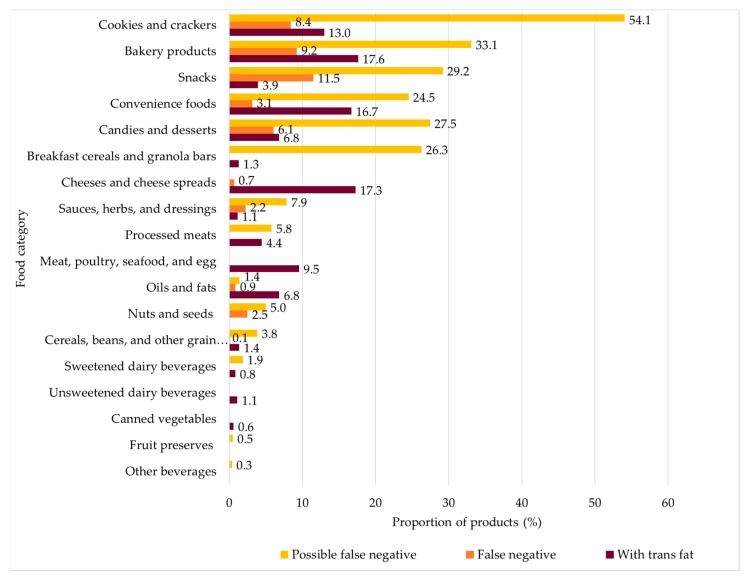
The proportion of products with trans fat content declared on the nutrition facts panel, false negatives, and possible false negatives by food category. Note: All the products of the sample were classified in the four groups related to the presence of trans fats (*n* = 11,434). Food categories in which all items were classified in the “without trans fats” group were not shown in this figure.

Figure 1 shows the proportion of foods and beverages based on the presence of trans fats. Approximately 10% of the cookies and crackers, bakery products, and snacks were false negatives (i.e., they had specific sources of trans fats in the list of ingredients but declared no trans fat content on the nutrition facts panel). More than half of the cookies and crackers and approximately one-third of the bakery products and snacks were possible false negatives (i.e., they listed possible sources of trans fats in the list of ingredients and declared being free of trans fats on the nutrition facts panel).

**Figure 2:**
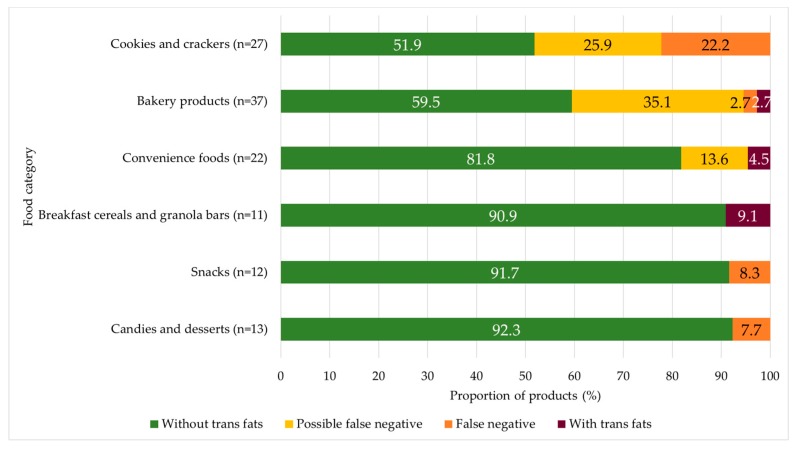
The proportion of products in the four categories related to the trans fat presence among the products displaying trans fat claims. Note: We displayed the six food categories with the highest proportion of trans fat claims in this figure. In other food categories, products with trans fat claims did not have trans fat ingredients. The exceptions were two products in cereal products (possible false negative) and two products in oils and fats (with trans fats). However, we decided not to show those groups in the figure since the total number of products with claims was very low.

Nutrition claims regarding trans fats were found in 4.6% of the assessed products. Bakery products had the highest proportion (21.1%) of foods with claims of the absence of trans fats, and in four other groups, claims were presented in around 10% of the products: breakfast cereal and granola bars (11.6%), convenience foods (9.2%), snacks (11.1%), and cookies and crackers (12.0%) (Table 3). Most of the foods with claims had no ingredients that were possible sources of trans fats in the list of ingredients. However, we found trans fat ingredients in 24.4% of the 160 products with claims. This proportion was higher in cookies and crackers and bakery products, in which almost half of the products could present some trans fat ingredient (Figure 2).

## 4. Discussion

Most of the Brazilian packaged foods and beverages did not report the presence of trans fats in the list of ingredients or on the nutrition facts panel. However, our results showed that industrially-produced trans fats may still be present in almost one-fifth of the products. Four percent of the evaluated products presented hydrogenated fats or oils. Furthermore, 15% of food and beverages had at least one ingredient that might be a source of trans fats, but that do not allow for a definitive assessment of their presence. Specific and unspecific sources of trans fats were also identified in foods with claims about their absence.

Although the content of trans fats in Brazilian food products has been shown to be reduced after the implementation of legislation on food labeling [31] and voluntary reduction agreements in the food industry [32], more recent evidence on this topic is lacking in the country. A 2010 study of food labels of products available in a supermarket in Santa Catarina, located in the Southern region of the country, found trans fat terms in more than half of the products. However, only 20% of the products declared trans fats on the nutrition facts panel [3]. Our sample included a larger number of products and food categories, but we found similar results for some categories, such as bakery products and cookies and crackers. Another study, conducted during the period 2014–2015 in Rio de Janeiro, in the Southeastern region of Brazil, evaluated some of the most consumed processed foods, including vegetable oils, margarine, biscuits, snacks, and ice cream, and found a drop in trans fat content in those products compared with similar research made in 2003, prior to the regulation. However, this drop in trans fats has not been observed in all products, and one of the samples of biscuits presented values as high as 12.9 g per 100 g [31].

Systematic reviews about the effectiveness of policies for reducing trans fats found that mandatory food labeling and voluntary self-regulation, the two measures adopted in Brazil, resulted in a decrease of trans fat levels in the food supply, but they are not as effective as national bans [33,34]. While the restriction led to elimination of trans fat in Denmark, in Canada, where mandatory labeling and voluntary limits were also implemented, some categories like bakery products and restaurant food remained with trans fats in a portion of the products [34]. Voluntary agreements depend on the extent to which the industry is willing to agree to and comply with the arrangement [35]. In Brazil, we have no official data on the progress obtained with the measure. Also, the information available on the total amount of trans fats withdrawn from the market was presented by the association of the food industries, without details on the applied methodology [32]. In the current Brazilian scenario, some products with trans fats continue to be sold and they usually are cheaper than a trans fat free option [36,37], which represents a disadvantage for lower-income consumers. Food labeling, on the other hand, depends on health literacy for consumers to use and understand it. Recent work conducted in Brazil showed that consumers use food labels to check nutrient content and ingredient information, but the format and the technicality of the labels often made the information inaccessible, particularly for those of low socioeconomic status [38]. Moreover, while it is an effective effort to influence consumer purchasing and product reformulation, mandatory labeling seems to have a limited long-term effect [39].

The scientific basis for the establishment of the cutoffs for the inclusion of trans fat content on nutrition facts panel and nutrition claims are not declared in the Brazilian legislation [14,17] and this is an issue that should be discussed. The current legislation allows food packages displaying zero content in products with 0.2 g of trans fats per serving. However, this amount corresponds to approximately 10% of the daily limit recommended by WHO (less than 1% of the total energy intake is equal to less than 2.2 g per day for a 2000-calorie diet) [9]. In comparison, the same cutoff of 0.2 g per serving is adopted for saturated fats, but this limit represents only 1% of the 22 g considered as a reference daily value for a 2000-calorie diet [14]. In addition, for some UPP, particularly those with trans fat ingredients, such as biscuits, chocolates, and snacks, the consumed serving sizes by Brazilian population are more than three times the declared serving size [16]. This means that the consumption of trans fats can exceed the upper recommended limit even when consumers choose products declared as trans fat free. Biochemical analyses of breads, cookies, oils, margarine, sauces, snacks, chocolates, cakes, and popcorn purchased in supermarkets located in a city of São Paulo found that most of the products complied with the legislation. However, only a small portion of the products that were declared as being free of trans fats on the nutrition facts panel did not have any trans fats in their composition (11 products out of 189—or 6%) [40].

Another important aspect of food labeling is the lack of standardization in the terms used in the list of ingredients, which may create confusion among consumers. Silveira et al. (2013) [3] identified that more than 20 ingredients may be a source of trans fats in Brazilian food products, and approximately half of those ingredients do not allow consumers to determine if trans fats are contained within the product. In this study, conducted in 2010, the majority of products with ingredients that are potential sources of trans fats presented imprecise terms, such as vegetable fat, vegetable cream, or margarine [3]. We found similar results which reinforce the need to establish and impose standardized names to be used in food labeling in the country to guarantee the right to information for consumers.

Regarding nutrition claims, as mentioned previously, the current Brazilian rules permit the use of messages that claim that the product is free of trans fats on products with trans fat content below 0.1 g per serving [17]. However, the presence of these claims on food products should be evaluated with caution. Positive messages about nutrition are used as marketing strategies by the food industry and have a selective focus, ignoring the presence of other unhealthy nutrients and encouraging the consumption of products with poor nutritional quality [41]. In this study, even foods with ingredients that are sources of trans fats showed trans fat free claims. Moreover, bakery products, cookies and crackers, candies and desserts, snacks, and convenience foods displayed the majority of trans fat claims. Many of the items in these food groups are classified as UPP [42] and should be avoided, according to the recommendations of the Brazilian Dietary Guidelines [43].

Imposing limits on the use of industrially-produced trans fats is the measure that has had the greatest impact on trans fat consumption in many countries [34], and it is currently under discussion in the regulatory process at the Brazilian agency [44]. This intervention has the advantage of not depending on individual actions and has the potential to positively impact the entire population [11]. Many countries have adopted measures restricting the use of trans fats in food production, such as Denmark, Hungary, and Norway, which have resulted in a drastic reduction in the use of such fats, revealing that these measures are feasible and effective public policies [45]. In Denmark, the first country in which restriction on the use of industrial trans fat was implemented, there was an estimated decrease in mortality attributable to CVD on average by about 14.2 deaths per 100,000 people per year in the three years that followed the policy [46]. Taking into account all the available evidence, recently, in April 2019, the European Union adopted a new regulation that must be effective until 2021, which limits the amount of industrially-produced trans fats in all foods to a maximum of two grams per 100 g of fat [47].

In 2008, the Pan American Health Organization (PAHO) launched the “Trans Fat Free Americas”, which recommended a limit of 5% of total fats from trans fats in industrialized food products and pointed to the need for legislative measures [48]. Countries in the Latin American region, such as Chile and Argentina, have already imposed a limit on trans fat use [49]. In 2015, in the United States (USA), the Food and Drug Administration (FDA) put partially hydrogenated oils, the main source of industrially-produced trans fats, on the list of substances that are not generally recognized as safe for use in food. Three years later, in 2018, nationwide bans were implemented on the use of this ingredient for the majority of the products in the USA [50]. The elimination of industrially-produced trans fats is a priority for WHO, which has recently launched a guide with strategic actions to be implemented in countries, including low- and middle-income countries in which the legislation regarding the use of industrially-produced trans fats is weaker [51].

Implementing policies that aim to restrict trans fats in the food supply results in the need for product reformulation, and the employed substitutes can be concerning as trans fats could be replaced with saturated fats to maintain the required or preferred solid fat content. However, a study conducted in 14 countries that aimed to compare foods with high and low content of trans fats showed that french fries, cookies, cakes, and microwave popcorn with low content of trans fatty acids had a higher content of saturated fats but also higher content of monounsaturated and polyunsaturated fatty acids [52]. The results of the aforementioned study indicated that, in different countries, trans fats were replaced with a combination of fats [52]. A separate study which investigated changes in the levels of trans and saturated fats in the United States from 1993 to 2009 found lower levels of both fats after reformulation in more than 90% of evaluated products [53]. Although any measure adopted to remove industrial trans fats would be expected to produce health benefits, WHO recommends the use of mono- and polyunsaturated fats as a replacement for trans fats rather than using animal fats and tropical oils that are high in saturated fat [9,51]. We suggest that the use of other industrially-produced fats, as interesterified fats, should be considered with caution because their health effects are not clear [54,55].

It is important to highlight that, initially, trans fat use by the food industry was considered a healthier option in relation to animal fat, due to the presence of unsaturation and its vegetable origin, but it is currently known that its consumption negatively impacts lipid metabolism, causing detrimental effects on the heart health [56]. The use of interesterified oils makes it possible to obtain similar sensory characteristics in food products without trans fat content, but the process results in a modification in the arrangement of fatty acids on the glycerol backbone and its influence on lipid metabolism is not understood [54,55]. Given the lack of scientific evidence on the health effect of consumption of interesterified fat, there is a need for research that contributes to the understanding of the effects of such fats on health to ensure the safety of its use and clarify the risks related to its consumption. The focus on the reformulation of products should not be the final goal in the discussion to promote healthier habits since recommendations made based only on the nutrients have been shown ineffective in dealing with current nutritional problems [56,57].

This study has some limitations. Our data source was the labels of food products marketed in the country and, to classify the product by the presence of trans fats, we used the information available in the list of ingredients and on the nutrition facts panel without conducting laboratory analyses, which may have introduced bias in our findings. Another limitation was the use of a random subsample to evaluate nutrition claims. Nonetheless, we were cautious in selecting our subsample, we included a large number of products, and we did not find any statistically significant differences in the proportion of foods in each food category or on the average content of nutrients, including trans fats, when we compared our subsample with the total sample of the assessed Brazilian packaged foods (data not shown). As a strength, we attempted to include all available packaged foods and beverages in the five largest food retailer chains in the country. We also took advantage of the INFORMAS claims taxonomy, which was developed to standardize the classification of the different health-related labeling components in different countries, to assess the presence of claims [29].

In conclusion, despite advances in recent years regarding the use of trans fats in Brazil, important issues concerning the presence and information about trans fats remain. In food labeling, there is a possibility that products are declared as trans fat free even with the presence of ingredients that are sources of trans fats. Another related issue is the deficiency of standardized terms in the list of ingredients. The lack of accurate information on packages can misinform consumers and lead to excessive consumption of trans fats without knowing. Food labeling with clear and adequate messages is a citizen’s right, as was established by the Brazilian Consumer Defense Code (Código de Defesa do Consumidor) [58]. In the case of trans fats, which are proven to cause significant health damage, the discussion on regulation must advance towards the restriction of industrial use. Trans fat limits should be imposed from the raw material used in the food industry, and no oils or fats with a high content of trans fats should be available to be used in Brazilian food products. This measure could benefit the entire population and aid inspection by competent agents. We believe our paper contributes to advancing the topic on the use of trans fats in Brazil and could be used to inform policymakers who are discussing the new regulation in the country, and also as a source of information for media and the general public about the products available in our supermarkets, aiming to increase public awareness of the need for policy options that supports better food environments.

## Figures and Tables

**Table 1:** Keywords used to identify products with trans fats in the list of ingredients.

Type of Term	Searched Items
Specific	Partially hydrogenated fat, hydrogenated vegetable oil, hydrogenated.
Unspecific	Vegetable fat, margarine, vegetable cream.

Adapted from Silveira et al., 2013 [3].

**Table 2:** Number and proportion of packaged foods with sources of trans fat in the list of ingredients and with trans fats declared on the nutrition facts panel.

Food Category	*n*	List of Ingredients						Nutrition Facts Panel					
		None		Specific		Unspecific		Zero or Insignificant		Above Zero		Not Informed	
		*n*	%	*n*	%	*n*	%	*n*	%	*n*	%	*n*	%
Breakfast cereals and granola bars	308	225	73.1	2	0.6	81	26.3	304	98.7	4	1.3	0	0.0
Bakery products	595	244	41.0	123	20.7	228	38.3	490	82.4	105	17.6	0	0.0
Convenience foods	795	477	60.0	48	6.0	270	34.0	651	81.9	133	16.7	11	1.4
Unsweetened dairy beverages	181	181	100.0	0	0.0	0	0.0	179	98.9	2	1.1	0	0.0
Sweetened dairy beverages	483	472	97.7	0	0.0	11	2.3	476	98.6	4	0.8	3	0.6
Snacks	356	203	57.0	45	12.6	108	30.3	340	95.5	14	3.9	2	0.6
Cookies and crackers	747	185	24.8	113	15.1	449	60.1	638	85.4	97	13.0	12	1.6
Canned vegetables	345	345	100.0	0	0.0	0	0.0	330	95.7	2	0.6	13	3.8
Oils and fats	351	338	96.3	7	2.0	6	1.7	325	92.6	24	6.8	2	0.6
Sauces, herbs, and dressings	801	720	89.9	18	2.2	63	7.9	785	98.0	9	1.1	7	0.9
Coffee and tea	94	94	100.0	0	0.0	0	0.0	84	89.4	0	0.0	10	10.6
Candies and desserts	1220	765	62.7	100	8.2	355	29.1	1132	92.8	83	6.8	5	0.4
Cereals, beans, other grain products	735	698	95.0	2	0.3	35	4.8	717	97.6	10	1.4	8	1.1
Packaged fruits and vegetables	907	907	100.0	0	0.0	0	0.0	841	92.7	0	0.0	66	7.3
Meat, poultry, seafood, and eggs	577	577	100.0	0	0.0	0	0.0	518	89.8	55	9.5	4	0.7
Sugar and other nonnutritive sweeteners	106	106	100.0	0	0.0	0	0.0	106	100.0	0	0.0	0	0.0
Processed meats	810	759	93.7	1	0.1	50	6.2	769	94.9	36	4.4	5	0.6
Fruit juices	150	150	100.0	0	0.0	0	0.0	150	100.0	0	0.0	0	0.0
Nectars	160	160	100.0	0	0.0	0	0.0	160	100.0	0	0.0	0	0.0
Fruit-flavored drinks	220	220	100.0	0	0.0	0	0.0	220	100.0	0	0.0	0	0.0
Carbonated beverages	106	106	100.0	0	0.0	0	0.0	106	100.0	0	0.0	0	0.0
Other beverages	286	285	99.7	0	0.0	1	0.3	281	98.3	0	0.0	5	1.7
Nuts and seeds	80	74	92.5	2	2.5	4	5.0	79	98.8	0	0.0	1	1.3
Cheeses and cheese spreads	607	594	97.9	11	1.8	2	0.3	502	82.7	105	17.3	0	0.0
Fruit preserves	414	412	99.5	0	0.0	2	0.5	410	99.0	0	0.0	4	1.0
**Total**	**11,434**	**9297**	**81.3**	**472**	**4.1**	**1665**	**14.6**	**10,593**	**92.6**	**683**	**6.0**	**158**	**1.4**

**Table 3:** Number and proportion of packaged foods with trans fat claims.

Food Category	n Subsample	Presence of Claims			
		*n*	%	95% CI	
Bakery products	175	37	21.1	15.7	27.8
Cookies and crackers	225	27	12.0	8.4	16.9
Breakfast cereals and granola bars	95	11	11.6	6.5	19.8
Snacks	108	12	11.1	6.4	18.6
Convenience foods	238	22	9.2	6.2	13.7
Fruit preserves	136	7	5.1	2.5	10.4
Candies and desserts	365	13	3.6	2.1	6.0
Oils and fats	106	3	2.8	0.9	8.5
Sauces, herbs, and dressings	262	7	2.7	1.3	5.5
Cereals, beans, other grain products	230	6	2.6	1.2	5.7
Processed meats	257	6	2.3	1.1	5.1
Nectars	50	1	2.0	0.3	13.1
Packaged fruits and vegetables	261	5	1.9	0.8	4.5
Other beverages	77	1	1.3	0.2	8.8
Meat, poultry, seafood, and eggs	171	2	1.2	0.3	4.6
Unsweetened dairy beverages	56	0	0.0	0.0	0.0
Sweetened dairy beverages	149	0	0.0	0.0	0.0
Canned vegetables	110	0	0.0	0.0	0.0
Coffee and tea	30	0	0.0	0.0	0.0
Sugar and other nonnutritive sweeteners	37	0	0.0	0.0	0.0
Fruit juices	53	0	0.0	0.0	0.0
Fruit-flavored drinks	63	0	0.0	0.0	0.0
Carbonated beverages	35	0	0.0	0.0	0.0
Nuts and seeds	22	0	0.0	0.0	0.0
Cheeses and cheese spreads	180	0	0.0	0.0	0.0
**Total**	**3491**	**160**	**4.6**	**3.9**	**5.3**

## Appendix A

**Table A1:** Description of food categories.

Food Category	Foods Included
Breakfast cereals and granola bars	Corn flakes, flavored oats, infant cereals, granolas, mueslis, granola bars, porridges, mix of cereals and fruits.
Bakery products	Breads, toasts, and cakes, including powders.
Convenience foods	Ready meals, French fries to frying, instant rice, instant noodles, instant soups, instant mashed potatoes, filled pastas, pizzas, pies, sandwiches, baby foods.
Unsweetened dairy products	Natural yogurts, milks, milk powders, milk compounds. Only products without sugar or nonnutritive sweeteners.
Sweetened dairy products	Yogurts, flavored milks, fermented milk, milk compounds, dairy beverages.
Snacks	Salted peanuts, salty snacks, potato chips, potato sticks, microwave popcorn (all flavors).
Cookies and crackers	Sweet and salty biscuits and cookies.
Canned vegetables	Canned beans and vegetables.
Oils and fats	Oils, margarines, butters, milk creams, vegetal fats.
Sauces, herbs, and dressings	Sauces, mayonnaises, herbs, catchup, salad dressings.
Coffee and tea	Coffees and herbs to prepare tea.
Candies and desserts	Chocolates, boiled sweets, flavored milk powder, jellies, cocoa powder, syrups, chocolate spreads, chewing gum, condensed milk, marshmallows, ice creams. Including versions with nonnutritive sweeteners.
Cereals, beans, other grain products	Beans, flours, rice, corns, pastas.
Packaged fruits and vegetables	Fruits and vegetables in natura, frozen fruit pulp, frozen vegetables.
Meat, poultry, seafood, and eggs	Meat, poultry, seafood, and egg, including chilled and frozen products.
Sugar and other nonnutritive sweeteners	Sugar, honey, nonnutritive sweeteners.
Processed meats	Burgers, sausages, canned fish, smoked meats, seasoned meats, salted meats, hams, salami, meat pates.
Fruit juices	Products declared as juices, fruit juices without water or added sugar, coconut water.
Nectars	Nectars made with juice and added water and/or sugar.
Fruit-flavored drinks	Fruit drink powder, fruit punch concentrate, fruit-flavored beverages.
Carbonated beverages	Sodas. Including diet and light versions.
Other beverages	Plant-based beverages, ready-to-drink teas, isotonic drinks, coconut milk.
Nuts and seeds	Nuts and seeds, including salted nuts.
Cheeses and cream cheeses	Cheeses, and cream cheeses.
Fruits preserves	Fruit jellies, fruit preserves, canned fruits, dried fruits, fruits sorbet.
